# Daily Quantity of Infant Leg Movement: Wearable Sensor Algorithm and Relationship to Walking Onset

**DOI:** 10.3390/s150819006

**Published:** 2015-08-04

**Authors:** Beth A. Smith, Ivan A. Trujillo-Priego, Christianne J. Lane, James M. Finley, Fay B. Horak

**Affiliations:** 1 Division of Biokinesiology and Physical Therapy, University of Southern California, Los Angeles, CA 90089-9006, USA; E-Mails: itrujill@usc.edu (I.A.T.-P.); jmfinley@usc.edu (J.M.F.); 2 Department of Preventative Medicine, Division of Biostatistics, Keck School of Medicine, University of Southern California, Los Angeles, CA 90089-9234, USA; E-Mail: christianne.lane@med.usc.edu; 3 Department of Neurology, Oregon Health & Science University and Portland Veterans Affairs Medical Center, Portland, OR 97239-3098, USA; E-Mail: horakf@ohsu.edu

**Keywords:** infant, movement, growth and development, wearable sensors

## Abstract

*Background*: Normative values are lacking for daily quantity of infant leg movements. This is critical for understanding the relationship between the quantity of leg movements and onset of independent walking, and will begin to inform early therapy intervention for infants at risk for developmental delay. *Methods*: We used wearable inertial movement sensors to record full-day leg movement activity from 12 infants with typical development, ages 1–12 months. Each infant was tested three times across 5 months, and followed until the onset of independent walking. We developed and validated an algorithm to identify infant-produced leg movements. *Results*: Infants moved their legs tens of thousands of times per day. There was a significant effect of leg movement quantity on walking onset. Infants who moved their legs more walked later than infants who moved their legs less, even when adjusting for age, developmental level or percentile length. We will need a much larger sample to adequately capture and describe the effect of movement experience on developmental rate. Our algorithm defines a leg movement in a specific way (each pause or change in direction is counted as a new movement), and further assessment of movement characteristics are necessary before we can fully understand and interpret our finding that infants who moved their legs more walked later than infants who moved their legs less. *Conclusions*: We have shown that typically-developing infants produce thousands of leg movements in a typical day, and that this can be accurately captured in the home environment using wearable sensors. In our small sample we can identify there is an effect of leg movement quantity on walking onset, however we cannot fully explain it.

## 1. Introduction

In the 1970s and 80s, Esther Thelen and colleagues described the developmental trajectory of infants’ spontaneous leg movements across the first year of life and explained how alternating kicking is a precursor to walking in typically-developing infants [1,2,3,4,5]. They did not, however, quantify how many leg movements an infant makes in a day or how much leg movement practice is necessary in order to learn to walk. While we know that typically-developing toddlers take approximately 9000 steps per day while learning to become skilled walkers [6,7], we do not know how much leg movement practice is necessary in order for walking to emerge.

Following Thelen’s seminal work, cross-sectional studies have shown that leg movements are different in infants who are at risk for delayed walking onset. Researchers have shown that kicking has altered characteristics in infants born preterm [8] and in infants with periventricular brain injuries [9,10,11], very low birth weight [12], Down syndrome [13,14], and myelomeningocele [15,16]. An inability to dissociate intralimb joint couplings during kicking, for example, can be observed as early as one month of age in preterm infants with white matter disorder [10], while in other work less organized spontaneous leg movements (indicating less adaptability) in infants with myelomeningocele at 3, 6, and 9 months of age were correlated with a later age of walking onset [16].

Frequency of kicking and movement during infancy are related to the attainment of independent walking, but the relationship is not clear. In infants with Down syndrome, Ulrich and Ulrich [13] showed that a higher frequency of kicking, but not overall movement, between 4 and 6 months of age was significantly correlated with an earlier age of onset of walking. The control groups (chronologically age-matched and motor-age matched according to developmental skill level) showed different relationships between kicking frequency and walking onset, however. In the chronologically age-matched control group, both frequency of kicking and overall movement were significantly correlated with earlier walking onset. In the motor age-matched control group, neither frequency of kicking nor overall movement was correlated with walking onset. Jeng and colleagues [17] found that an increased kicking frequency at 4 months corrected age in very low birth weight infants was correlated with later attainment of walking. It could be that correlations between kicking frequency, movement frequency and walking onset are positive for some groups and negative for others. Another explanation is that the 5–8 min of movement observed in these studies [13,17] is not sufficient to accurately capture the relationships of interest. Infant behavior is highly variable and affected by context, thus a snapshot of behavior may not reflect the true relationship [18]. Currently, there is no benchmark for the daily quantity of leg movements generated by typically-developing infants. This information is critical for understanding the relationship between quantity of leg movements and onset of walking.

In this study, we used inertial movement sensors to record full-day leg movement activity from infants with typical development. Each infant was tested three times across 5 months, and followed until the onset of walking. We developed an algorithm to determine the number of leg movements performed daily, and related this to onset of walking. Our algorithm counted a separate leg movement each time the infant paused or changed direction of the leg. By recording full-day data we are able to directly measure the number of leg movements made in a day in the infants’ natural environment. Our results will provide the first step toward developing developmental norms.

## 2. Experimental Section

### 2.1. Participants

Twelve infants with typical development (eight female, four male) in the Portland, OR metropolitan area participated. Infants started the study between 1 and 8 months of age, and were tested three times with 2 months between visits. They were followed until the onset of independent walking. The study was approved by the Institutional Review Board of Oregon Health & Science University. Parents signed an informed consent form for their infants’ participation.

### 2.2. Data Collection

**Figure 1:**
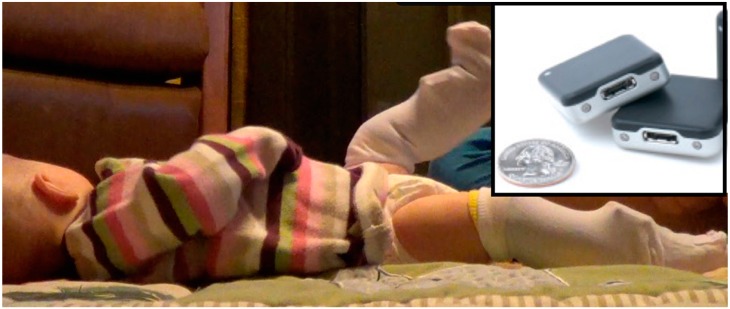
Three-month-old infant wearing sensors on the front of each ankle. Inset: Inertial sensors (Opals; APDM Inc., Portland, OR, USA) are synchronized, wireless, small and lightweight. They measure 48.4 × 36.1 × 13.4 mm (L × W × H) and weigh just under 22 g each. Opals are shown with a standard U.S. quarter for size reference.

At each visit, we administered the Alberta Infant Motor Scale (AIMS) to quantify motor development status [19] and measured weight, length, and head circumference. We placed inertial movement sensors (Opals, APDM, Inc., Portland, OR, USA) on each leg using knee socks (see Figure 1). Sensors were firmly attached to the bottom layer knee sock just proximal to the ankle joint using Velcro^®^, and covered by a second, more pliable knee sock. They collected actively synchronized tri-axial accelerometer and gyroscope data at 20 samples per second. Data were stored on each sensors’ internal memory, and downloaded following the collection. The visit always took place in the morning, and the sensors remained in place until bedtime, 8–13 h later, when the parents removed them. They were instructed to go about their normal activities and record the time, position and activity of their infant as best they could in an activity log, with particular attention to time in cars or strollers where background movement was recorded. In addition, during the morning visit, spontaneous movement video data were recorded at 30 frames per second for 5 min, while the infant wore the sensors. Infants were awake, alert and content during video recording. Infants 6 months and younger were recorded in supine. Infants 7 months and older were recorded in supported standing to prevent them from rolling or crawling away during the recording. Videos were obtained to provide gold standard observation of leg movements. Parents were contacted in follow-up to determine the onset of independent walking (three steps without assistance).

### 2.3. Data Analyses: Algorithm Development

A threshold-based algorithm was developed to differentiate leg movements from non-infant produced movement or noise. Algorithm development was achieved through manual “training” on a subset of data. We compared the sensor data to synchronized video data and varied the parameters of acceleration and angular velocity thresholds and pattern requirements (described below) until movements were accurately identified. Our goal was to create a robust algorithm to describe the pattern of acceleration and angular velocity produced by infant leg movements as identified in the video. A leg movement was defined as a movement in which the limb changed position in space by the infant’s effort and could be observed visually, in real time. We then proceeded with an automated validation phase on 23/36 files where the algorithm automatically quantified movements, which we then compared to the gold standard of manual counting through video coding. Video coding is the gold standard for identifying presence and type of infant leg movements. It should be noted, however, that previous analysis has focused on identifying kicks or other specifically defined types of leg movements, not all leg movements as we did here [13,17,20].

From the sensors, the tri-axial acceleration (m/s^2^) and tri-axial angular velocity (rad/s) signals at 20 Hz were analyzed with custom Matlab programs. We calculated the magnitude of the acceleration vector (Equation (1)) and the magnitude of a vector composed of each of the angular velocity components (Equation (2)) at each time point from calibrated data for each leg for the time period the sensors were worn. We used this equation as movement can cause acceleration or rotation on any, or all, of the axes and our detection algorithm needed to be sensitive to movements in any direction. Next we used the Matlab detrend function to remove linear drift in the signals. This set the acceleration baseline to 0:

(1) $${\text{Accel}}_{mag}=\sqrt{a_{x}^{2}+a_{y}^{2}+a_{z}^{2}}$$

(2) $${\text{Angular velocity}}_{\text{mag}}=\sqrt{{\text{ω}}_{\text{x}}^{2}+{\text{ω}}_{\text{y}}^{2}+{\text{ω}}_{\text{z}}^{2}}$$

The start of a movement was defined as simultaneous acceleration above a magnitude threshold and angular velocity greater than 0. The end of a movement was defined after two crossings of the acceleration baseline from different directions. This defined a separate movement each time the infant paused or changed direction of the leg. For example, an upright step consisting of simultaneous flexion of the hip and knee followed by simultaneous extension of the hip and knee would be counted as two movements, a supine kick consisting of simultaneous flexion of the hip and knee followed by simultaneous extension of the hip and knee would be counted as two movements, and a supine leg movement series of flexion of the hip with a straight knee followed by flexion of the knee, extension of the knee, and then abduction/external rotation to bring the leg to the ground would be counted as 4 movements.

**Figure 2:**
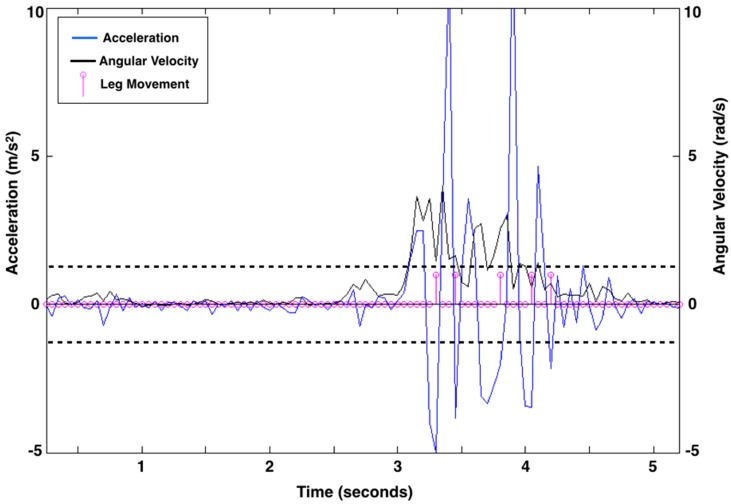
Leg movement count from 5 s of representative acceleration (linear acceleration as m/s^2^; blue line) and rotation (angular velocity as rad/s; black line) data from the right leg of a 3-month-old infant. Pink circles along the baseline represent data points at 20 samples per second. There are five leg movements (shown as pink lines with circles at the end of a movement) identified by the algorithm. The acceleration thresholds, represented by dashed black lines, were 1.274 m/s^2^ above baseline and 1.257 m/s^2^ below baseline for the right leg of this child at this visit. Acceleration thresholds were calculated uniquely for each leg at each visit.

**Figure 3:**
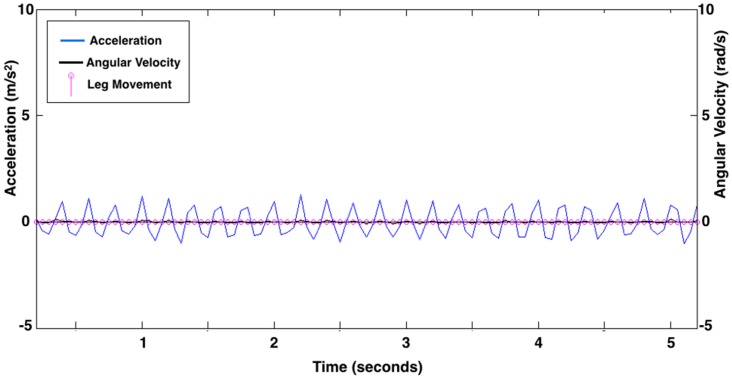
Leg movement count from 5 s of representative acceleration (linear acceleration as m/s^2^; blue line) and rotation (angular velocity as rad/s; black line) data showing the movement of a mechanical swing recorded while the infant was asleep. There are no leg movements identified by the algorithm. Pink circles along the baseline represent data points at 20 samples per second.

For each data collection, there was a clear visual difference between acceleration due to leg movement and acceleration due to noise, however the actual values varied across collections. The smallest acceleration peaks an infant produced that reflected movement, and not noise, were consistent across a data collection but varied between 1.00 to 3.25 m/s^2^ absolute magnitude of acceleration between collections. The acceleration threshold was determined for each collection by finding, separately, all acceleration peaks with a magnitude of 1.00 to 3.25 m/s^2^ (positive peaks above baseline and then negative peaks below baseline) for each leg. We calculated the mean and standard deviation of the acceleration of the peaks and subtracted the standard deviation from the mean to set the positive acceleration threshold. We similarly defined a negative threshold. The thresholds were created using data from the entire visit and effectively separate acceleration due to leg movement and acceleration due to noise for each infant’s unique acceleration profile at each visit. Figure 2 shows the leg movement count from 5 s of exemplar data. Figure 3 shows data from an infant asleep in a mechanical swing. The algorithm did not count any leg movements here because the data did not meet the definition of a leg movement.

### 2.4. Data Analyses: Algorithm Validation

From the video data, one observer identified separate movements each time the infant paused or changed direction of the leg (consistent with the algorithm definition). The occurrence of a movement was identified in real time, with frame-by-frame analysis used to confirm pauses or changes in direction. We selected 20 s when the infant was moving and compared the number of movements counted by the algorithm to the number identified in the video.

### 2.5. Statistical Analyses

We used linear mixed effects models (SPSS Statistics for Mac, Version 22.0. IBM Corporation, Armonk, NY, USA) to test for an effect of movement rate (average of right and left legs at each visit) on walking onset (age in days at the onset of three independent steps), adjusting for repeated measurements of each infant. We acknowledge that there are likely many factors impacting the onset of walking, including length/weight/strength ratios, motivation, postural control, and parenting styles. Because our main goal was to assess the role of leg movement quantity on the onset of independent walking, we first examined the effect of movement rate only. 

**Table 1:** Infant anthropometric and developmental scale measurements, by visit.

Infant	Visit	Age at Visit (Months)	Albert Infant Motor Scale (Raw Score)	Weight (kg)	Body Length (cm)	Head Circumference (cm)
a	1	6	29	6.4	61.0	43.0
	2	8	39	8.2	64.5	45.0
	3	10	53	8.7	68.6	45.0
b	1	1	5	4.2	55.0	37.0
	2	3	13	6.0	64.8	40.5
	3	5	21	7.6	66.0	41.2
c	1	7	32	8.2	64.8	35.5
	2	9	51	9.0	66.0	47.0
	3	11	53	9.3	71.1	48.0
d	1	8	31	8.9	70.0	45.0
	2	10	41	9.1	74.0	46.0
	3	12	51	9.9	76.2	47.0
e	1	2	7	4.7	56.5	40.0
	2	4	17	6.6	62.5	42.5
	3	6	26	7.5	67.3	44.0
f	1	3	8	3.8	59.0	38.0
	2	5	15	6.2	63.5	39.0
	3	7	27	6.8	63.5	41.5
g	1	8	26	9.1	73.0	46.0
	2	10	38	9.7	74.0	46.0
	3	12	52	10.0	76.2	49.0
h	1	7	23	7.3	72.1	45.7
	2	9	34	8.3	73.0	47.0
	3	11	42	9.4	75.0	48.0
i	1	3	8	6.4	59.5	41.0
	2	5	24	7.2	64.8	43.0
	3	7	42	7.7	68.6	44.5
j	1	5	16	6.4	60.0	40.0
	2	7	29	7.2	64.8	42.0
	3	9	35	7.5	66.0	42.7
k	1	5	22	8.3	65.0	45.5
	2	7	30	9.5	71.0	47.0
	3	9	50	10.0	71.0	48.0
l	1	2	9	6.0	60.0	39.0
	2	4	21	7.7	67.5	42.3
	3	6	34	8.7	71.1	44.0

Next, to examine the influence of age, overall body size, and varying developmental rate, we ran separate models adjusting for age (in months), percentile length-for-age (determined from Table 1 and standardized growth charts) [21], and developmental level (raw AIMS score) at each visit. These covariates were modeled independently given the small sample size. In all models we used visit as a repeated measure, with a diagonal covariance matrix, and α = 0.10.

## 3. Results and Discussion

### 3.1. Algorithm Validation

Analysis of 460 s across 23 videos resulted in the visual identification of 636 movements across the right and left legs. The algorithm identified 634 movements, making 48 mistakes while identifying 588 movements correctly. The algorithm over-counted (false positives) by 23 movements and under-counted (false negatives) by 25 movements. The sensitivity was 92%.

**Figure 4:**
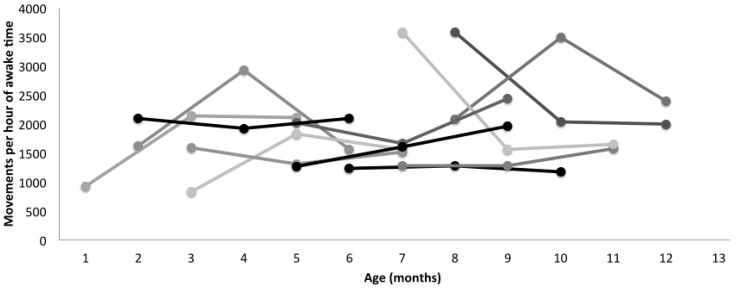
Average leg movement rate of infants, calculated as number of movements per hour of awake time (average of right and left legs). Each line represents a typically-developing infant across three visits. Lines are shown for ease of viewing and are not meant to imply a linear fit across visits. Data were collected at three time points only, as indicated by a round marker for each infant. Data are plotted by chronological age.

Based on the duration of the videos and the number of movements identified, we calculated an average rate of 1.4 movements/s. As shown in Figure 4, the low end of the movement rate for full-day data was around 1000 movements/3600 s of awake time, or 0.3 movements/s, while the upper end of the range was just under 4000 movements/3600 s of awake time, or 1.1 movements/s. Our video sections used for validation, selected during periods of infant movement, are consistent with the upper range of movement rate observed.

### 3.2. Walking Onset

Walking onset occurred for all infants between 9 and 18 months, as expected in typical development. See Table 2 for age at walking onset.

### 3.3. Quantity of Leg Movements

Leg movements were quantified for each leg for the period of time the sensors were worn, from 8–13 h (see Table 2).

**Table 2:** Infant movement characteristics, by visit.

Infant	Walk Onset (Age in Days)	Visit	Age at Visit (Months)	Awake Time (h)	Movements Per Day		Movement Rate *		
					(Left Leg)	(Right Leg)	(Left Leg)		(Right Leg)
a	320	1	6	11.0	12,899	14,348	1173		1304
		2	8	9.50	12,695	11,669	1336		1228
		3	10	7.50	8067	9407	1076		1254
b	291	1	1	11.0	10,039	10,262	913		933
		2	3	9.50	19,056	21,599	2006		2274
		3	5	8.75	18,604	18,160	2126		2075
c	329	1	7	9.75	12,158	12,758	1247		1309
		2	9	7.25	9236	9369	1274		1292
		3	11	8.00	13,570	11,516	1696		1440
d	481	1	8	5.25	19,448	18,046	3704		3437
		2	10	8.50	14,527	19,986	1709		2351
		3	12	8.00	16,868	15,068	2109		1884
e	519	1	2	7.50	11,553	12,631	1540		1684
		2	4	8.50	24,168	25,645	2843		3017
		3	6	8.50	12,974	13,611	1526		1601
f	495	1	3	8.00	6502	6771	813		846
		2	5	7.75	12,963	15,434	1673		1991
		3	7	6.50	9403	10,938	1447		1683
g	461	1	8	8.00	14,854	18,461	1857		2308
		2	10	7.50	25,904	26,536	3454		3538
		3	12	8.50	21,224	19,454	2497		2289
h	509	1	7	7.25	25,371	26,616	3499		3671
		2	9	6.75	9864	11,153	1461		1652
		3	11	7.00	11,836	11,315	1691		1616
i	376	1	3	10.00	15,371	16,350	1537		1635
		2	5	8.25	11,321	10,251	1372		1243
		3	7	6.75	10,076	10,340	1493		1532
j	426	1	5	10.00	20,137	20,289	2014		2029
		2	7	9.50	16,116	15,419	1696		1623
		3	9	7.50	18,893	17,594	2519		2346
k	426	1	5	9.75	10,928	13,768	1121		1412
		2	7	10.00	15,511	16,547	1551		1655
		3	9	8.00	15,684	15,726	1961		1966
l	288	1	2	9.50	17,494	22,242	1841		2341
		2	4	10.50	19,862	20,494	1892		1952
		3	6	9.25	19,722	18,986	2132		2053
Mean (Standard Deviation)					15,136 (4892)	15,799 (5060)		1828 (687)	1902 (678)

**Figure 5:**
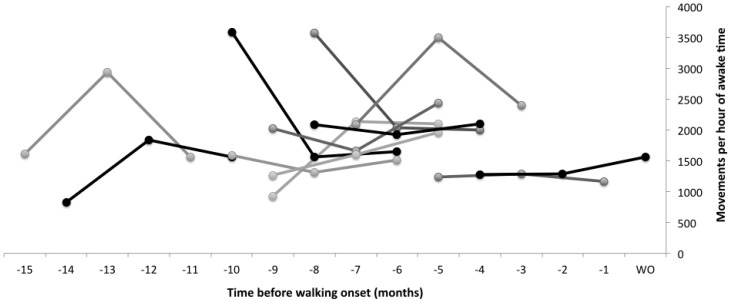
Average leg movement rate of infants, calculated as number of movements per hour of awake time (average of right and left legs). Each visit is shown as a round marker. Each line represents a typically-developing infant across three visits. Lines are shown for ease of viewing and are not meant to imply a linear fit across visits. Data were collected at three time points only, as indicated by a round marker for each infant. Data are plotted in relation to how many months before walking onset (WO) infants were assessed. Onset of walking occurred between 9 and 18 months chronological age.

To compare leg movement quantity across infants, who had different lengths of data collection and amounts of naptime, we calculated movement rate per hour of awake time. To do this, we determined time asleep (to the nearest 10 min) from the activity log and verified that the sensors were mostly still during the identified periods. In the event an infant woke briefly and moved, those movements were included in our count. Figure 4 shows average leg movement rate per hour of awake time for each infant, by chronological age. In addition to being tested at different chronological ages, infants were also at different developmental points when they were tested, as shown in Table 1. Figure 5 shows average leg movement rate per hour of awake time for each infant in relation to when they started walking independently. Some infants were tested close to walking onset, whereas others were months before walking when they were tested.

### 3.4. Relationship between Leg Movement Quantity and Walking Onset

Infants who moved more across a day walked significantly later than infants who moved less (β = 0.041, *p* = 0.05). This effect remained significant when adjusting for age (β = 0.036, *p* = 0.09), developmental level (β = 0.041, *p* = 0.05), or percentile length-for-age (β = 0.041, *p* = 0.07) at the time of the visit. The β coefficients for the effect of movement rate on walking onset reveal that for every 1000 movements more per hour of awake time infants walked, on average, 41 days later. When adjusting for age the effect is 36 days and when adjusting for developmental level or percentile length-for-age it remains 41 days.

## 4. Discussion

We were able to accurately identify the number of daily leg movements infants’ produced from wearable sensor data collected in their home environment while they went about their typical activities. There are two key points to our algorithm’s accuracy: (1) we set an acceleration threshold based on the unique movement characteristics of each infant, establishing a unique “noise” threshold based on the statistical properties of their movement; and (2) we require both acceleration and rotation to be present in order to count a movement, which ignores motion from cars, strollers and mechanical swings (where predominately linear acceleration is present).

We validated our algorithm against visually observed movements in video data, defining a separate leg movement each time the infant pauses or changes direction of the limb. This means that a leg movement, as counted by the algorithm, corresponds to what a visual observer would identify as a leg movement, and represents some change in the state of the nervous system to pause or move the limb. While we are able to exclude background motion produced by a stroller, car or mechanical swing, there is unavoidable error when the infant’s legs are moved by the parent (such as a diaper change or during dressing), however these activities are minimal in contrast to the large number of infant-produced movements. Further, infant-produced movements during handling are counted.

We have provided the accuracy of the algorithm to count movements, and specified how many it over and under counted by. An under count can happen, for example, when the algorithm counts two movements but the observer says there are three during a movement complex. We cannot determine specificity because there is no way for an observer to accurately count periods of no movement. There can be very small periods of no movement between two movements in a series, for example. The algorithm can measure, for example, 10 ms of no movement (two samples) between two movements, but an observer cannot. In this case, an observer could only count that there were two movements that occurred by visual discrimination (as would the algorithm). The goal of our algorithm is to quantify movements, as an observer would count them (not more or less), and the algorithm can do that quite well with only minor occurrences of over or under counting compared to the gold standard observer.

In regard to an effect of the sensors themselves on infant movement production, parents reported that their infants were not affected by wearing the sensors. Further, in a subset of seven infants, there was not a significant difference in leg movements produced in 4 min in supine when infants were wearing or not wearing the sensors (Repeated Measures Analysis of Variance); (*F*_1,13_ = 0.01, *p* = 0.92).

Wearable sensors provide an efficient way of having a portable device for quantifying full-day infant movement. Once norms have been established in a large enough sample of infants, there is potential application in early assessment of impaired neuromotor development and as an outcome measure of the effects of intervention. Having a valid algorithm for determining infant-produced leg movements from full-day data is the first step in being able to measure differences between populations or evaluating outcomes.

In our sample of 12 typically-developing infants, there was a significant effect of leg movement quantity on walking onset. Infants who moved their legs more walked later than infants who moved their legs less, even adjusting for age, developmental level or percentile length-for-age at the visit. The beta coefficients reveal that for every 1000 movements more per hour of awake time infants walked, on average, 41 days later, although it should be noted that all infants started walking independently within the typical expected range of 9–18 months. Further, we did not assess infant percentile length-for-age at the time of walking onset, only at the time leg movement data were collected. It will be of key importance to explore these data further, as what infants are doing when they are moving is as important, if not more important, than how much they are moving. It is expected, for example, that leg movement quantity would decrease when infants learn to sit independently as they would be using their legs to stabilize their sitting posture instead of moving them freely while in a supine position. Adjusting for developmental level in the statistical analysis does not allow us to understand an effect like this in such detail. As Figure 4 and Figure 5 show, the relationship between movement quantity and development are not simple to observe. Infants develop at different rates, and we need to collect data from a much larger sample in order to understand the relationship between movement quantity and type and developmental rates. Our algorithm will allow us to do so moving forward.

Moving forward, we will continue to explore the data to assess variability across days and months, patterns (unilateral *vs.* bilateral, in-phase *vs.* anti-phase), and types of limb movements (kicks *vs.* other). Our current results may indicate: (1) infants who are more efficient at learning move their legs less and walk earlier; or (2) what infants are doing (not reported here) is as important, or more important, than how much they are moving. Our results are consistent with previous researchers who found that increased frequency of kicking at 4 months corrected age in very low birth weight infants was correlated with later attainment of independent walking [17]. We would like to again point out that infant developmental rates are highly variable, and we will need a much larger sample to adequately capture and describe the effect of movement experience on developmental rate. Our algorithm defines a leg movement in a specific way (each pause or change in direction is counted as a new movement), and further assessment of movement characteristics are necessary before we can fully understand and interpret our finding that infants who moved their legs more walked later than infants who moved their legs less.

### Limitations

Our goal was to create and validate an algorithm to determine infant-produced leg movements from full-day wearable sensor data. Twelve infants is a small sample size, and although we were able to measure an effect of leg movement quantity on walking onset, we are not able to fully explain it. Our data were collected in one regional area; the sample does not reflect cultural or other variation in parenting practices. We collected data across different ages and developmental levels. We measured infant size at the data collection, but not at walking onset. Finding an effect of leg movement rate on walking onset in our small sample supports moving forward with an adequately-powered sample to use wearable sensors to capture and accurately describe this effect across a broad, representative population.

## 5. Conclusions/Outlook

In this paper we have shown that typically-developing infants generally produce tens of thousands of leg movements in a day, and that this can be accurately captured in the home environment using wearable sensors. Our results demonstrated a significant effect of leg movement quantity on walking onset, an effect that needs further exploration. We will use our results here to power an appropriately-sized study to create population norms for leg movement assessment in infants with typical development and assess infants at risk for developmental delay. In future work we will focus on describing how characteristics of leg movements beyond quantity change over time, using different types of analyses to explore patterns and types of movements produced as well as stability and predictability of movements across time. We will also explore options to further refine our detection algorithm using filtering and signal decomposition techniques such as Independent Component Analysis. Lastly, we will relate leg movement characteristics to functional developmental outcomes, and determine when significant differences are present between infants with typical development and infants with developmental delay.
